# Exploring Women’s Personal Experiences of Giving Birth in Gonabad City: A Qualitative Study

**DOI:** 10.5539/gjhs.v6n5p46

**Published:** 2014-05-09

**Authors:** Fariba Askari, Alireza Atarodi, Shirin Torabi, Mahdi Moshki

**Affiliations:** 1 Department of Midwifery, School of Nursing and Midwifery, Gonabad University of Medical Sciences, Gonabad, Khorasan Razavi, I.R.Iran; 2 Department of Basic sciences, School of Paramedical Sciences, Gonabad University of Medical Sciences, and Social Development & Health Promotion Research Center Gonabad, Khorasan Razavi, I.R.Iran; 3 Department of Public Health, School of Health And Social Development & Health Promotion Research Center, Gonabad University of Medical Sciences, Gonabad, Khorasan Razavi, I.R.Iran

**Keywords:** Childbirth, giving birth, midwifery cares, qualitative study

## Abstract

**Background:**

Women’s health is an important task in society. The aim of this qualitative study that used a phenomenological approach was to explain women’s personal experiences of giving birth in Gonabad city that had positive experiences of giving birth in order to establish quality cares and the related factors of midwifery cares for this physiological phenomenon.

**Methods:**

The participants were 21 primiparae women who gave a normal and or uncomplicated giving birth in the hospital of Gonabad University of medical sciences. Based on a purposeful approach in-depth interviews were continued to reach data saturation. The data were collected through open and semi-structured interactional in-depth interviews with all the participants. All the interviews were taped, transcribed and then analyzed through a qualitative content analysis method to identify the concepts and themes.

**Findings:**

Some categories were emerged. A quiet and safe environment was the most urgent need of the most women giving birth. Unnecessary routine interventions that are performed on all women regardless of their needs and should be avoided were considered such as: “absolute rest, establishing vein, frequent vaginal examinations, fasting and early Amniotomy”. All the women wanted to take part actively in their giving birth, because they believed it could affect their giving birth.

**Conclusion:**

We hope that the women’s experiences of giving birth will be a pleasant and enjoyable experience for all the mothers giving birth.

## 1. Introduction

Many phenomena in the world are common to most human societies for example, birth and death and they seem obvious and natural, but these natural phenomena can be affected by pathological processes. Then, health cares and treatments are needed to improve them; however there are control and treatment techniques in the world of modern medicine (Dahlberg, Berg, & Lundgren, 1993). Although, moment of giving birth is considered a happy stage in life and can have a positive impact on women and their mental status as well, but objectively it can leave harmful effects on their emotions and health (Murphy, Pope, Frost, & Liebling, 2003). The exploration of women’s experiences of giving birth is important to help them in their giving birth effectively. Giving birth is a painful and fearful process, however emotional support of those engaged in giving birth process can reduce laborers stresses and pains (Javanmanesh, Yekkeh yazdandoost, & Bastani, 2002), even if women’s mood is usually in a high level during the process of giving birth (Javadnoori, Afshari, Montazeri, & Latifi, 2008). Iranian women such as women in the other parts of the world wish and expect a healthy and successful giving birth phenomenon. When a process is assisted by the caregiver midwives, two basic questions must be into account: 1. Will that process improve maternal outcomes improvement? 2. Will that process improve neonatal outcomes improvements? Unfortunately, there are some techniques which are employed in normal process of women’s giving birth that not only have no benefit for maternal and neonatal outcomes improvement, but also potentially can create some risks for both of them (Robertson, 2008). When performing a treatment protocol, midwife or gynecologist should consider how to perform a routine care, since it can generate a negative attitude for the mothers. For example, performing a routine shaving technique can make the women more anxious, since in their opinions, the caregivers, apparently, wouldn’t be able to prepare them for surgery process. If women have a meeting with the midwife who provides care in giving birth, it can help women to fully understand the events of giving birth and feel somehow relax. For example, if she was not allowed to have a normal position for herself outside the bed, she would feel unable and couldn’t help herself in giving birth (Robertson, 2008). Routine interventions during giving birth are common in Taiwan such as perinea shaving, enema, episiotomy, electronic fetal monitoring, however, these interventions are not often needed and there is no evidence to show its usefulness for reduction of wounds and newborn infections. Routine episiotomy also has not any justified benefits to reduce the severity of perineal injuries and pelvic relaxation (Chen-Yu & Kuo-Gon, 2006). Historical studies showed that women have always been supported in giving birth, but in today’s world, in most countries that giving birth is performed in hospitals; women do not receive such kinds of supports. The studies performed on the women’s experiences of giving birth have shown that constant caring should be performed by midwives (Hodnett, Gates, Hofmeyr, & Sakala, 2007). During the last 30 years, cares during giving birth have not been improved much, however today this trend has shifted towards normal giving birth because of human and emotional aspects of giving birth; a mother is considered an active person and does not know giving birth as a mechanical process (Chen-Yu & Kuo-Gon, 2006) and this is one of the principles of mother-friendly hospitals in normal process of giving birth. The protocol is believed that mother and child have indigenous consciousness necessary for recognition of giving birth. Giving birth is a normal process and a trend should be performed through minimal medical intervention and such interventions must be performed only for the cases in which the mother or fetus life is at risk or in danger (Health Ministry of Iran, 2006). It is also important to survey how women feel about their experiences and what they need. Women’s opinions on the cares they need during different stages of giving birth and how the cares should be performed need to be taken into consideration. A qualitative study conducted in the United States on pregnant women showed that the majority of pregnant women expected their labor nurse to provide them physical comfort as well as emotional support along with routine nursing care such as monitoring the mother and baby. The authors logically asserted that, if nurses and hospitals try to fulfill women’s expectations of their birth experience, a greater chance will emerge to have positive childbirth experiences and to be more satisfied with their care (Tumblin & Simkin, 2001).

Also women’s acceptance and agreement on giving birth process can be effective and influence the process of giving birth; however, there is a little information on this idea, opinion and experience. Understanding of perceived cares from women’s views can increase midwifery staffs’ ability to perform their tasks more appropriately. Considering that pregnancy and giving birth services should not be based on the need of the service provider, but on the need of mother and child. Fear of pain on giving birth has become an epidemic in modern world among pregnant women. More than ever, women are frightened of giving birth and what might possibly go around. Then women, who are afraid of giving birth, need psychological support, obstetric guidance and education. The female’s body is designed to stop giving birth in unsafe situations, and the mother being in a heightened state of fear is a trigger for stopping the process. As it was said and understood, mothers have some experiences of their giving birth that can be a base and guide for their care providers to help them more easily and effectively and these experiences and also supports should not be neglected. Then, the present study was conducted with the aim of exploring women’s personal experiences of giving birth in Gonabad city.

## 2. Materials and Methods

A qualitative approach, specifically phenomenology was chosen because the purpose of the study was to explore and describe the meanings and concepts of the women’s experiences of giving birth. There are many interpretations of phenomenology and the philosophical beliefs of phenomenologists are different with each others. Since the aim of this study was to explore, describe and understand women’s experiences of giving birth, therefore the Husserlian phenomenological approach was used, because Husserl’s interest was in studying the phenomena as they are appeared through the consciousness of individuals. And also this qualitative approach was used in order to survey women’s experiences of giving birth with the aim of achieving a diverse picture of women’s experiences in giving birth. Then, 21 primiparae women with the age range of 19-29 years, who willingly took part in the study, were surveyed through in-depth interviews. Based on a purposeful sampling the interviews were continued to reach data saturation and no more necessary interviews were diagnosed. All the interviews were taped, transcribed and then analyzed by a qualitative content analysis method. The participants’ literacy and social class were excluded since their experiences of giving birth were the most intention of the study. A scientific process seems necessary to show women’s views or concepts of giving birth as they really emerge. In this study Husserl philosophic approach (1931, 1965) was used. In fact, willingness and attitude to the reality of experience based on understanding of reality and new ways have been proposed to perform giving birth cares.

### 2.1 Gonabad Hospital Maternity Ward

Gonabad city with population over 120,000 and an area of 10,000 square kilometers is located in south of Khorasan-e-Razavi Province, 260 km far from Mashhad. It is located in east longitude of 46-57 to 27-59 and latitude of 30-34 to 34-54 (Ramezani Awal Riabi & Atarodi, 2012). It has one hospital with some wards. In maternity ward approximately 200-250 women are treated for giving birth every month. It welcomes all people 24/7 and provides services for all of them, although there are some limitations and shortages that let not to perform some measures over there and for example: some non-evidence based practices are being used that some of them are 1- routine episiotomy, 2- not allowing companionship during labor, 3- use of procedures to speed up labor without indications, 4- restriction of mobility, 5- supine position for delivery, 7. Shortage of personnel midwives in the delivery ward more often, and 8- non-use of music and drug therapy and some other needed measures for decreasing pain. However some measures are conducted such as: routine enema, giving oral fluids, and use of active management of the third stage of labor

### 2.2 Ethical Considerations

The study was approved by the Ethics Committee of research of Gonabad University of medical sciences. The participants were orally informed about the study. All the participants were informed of the study objectives which were women’s experiences of giving birth and the participants were taught how to perform the in-depth interviews. Before the interviews, the participants’ written informed consent was obtained. The interviews were recorded on tapes and they were assured that the information would remain confidential and anonymous and the participants were also told that they would be allowed during the interviews to cancel or leave it if they would not like to continue their interviews at any stage. The interviews were conducted in a calm and silent room that was a special place in their own choice.

### 2.3 Procedure

In the present study, unstructured interviews were used for collecting data which allowed flexibility and made it possible for the researchers to follow the interests and thoughts of the participants.

This qualitative study with a purposeful sampling was performed; the study samples were 21 primiparae women with the age group of 19-29 years that had normal giving birth in Gonabad University of medical sciences’ hospitals without any giving birth-related complications that agreed to participate voluntarily in the study. We obtained written informed consent because our samples were human and the study was also approved by the Ethics committee of research. The data were collected by open and semi-structured in-depth interviews when the participants were referring to health care centers to receive their 2^nd^ postpartum cares about two weeks after their giving birth. Bracketing was performed by the interviewers before performing the interviews. The use of bracketing defends the validity and objectivity of interpretation against the self-interest of the researcher (Koch, 1995).

The interviews were audio-taped in a calm and silent room that was a special place and the women were also interviewed at the location of their own choice. The interviews were conducted in the form of a dialogue and the interviewees were encouraged to talk openly with no obstacle about their experiences of giving birth. The first question posed to each participant was to describe “how they generally experienced the first stage of their giving birth and what kind of cares they received”. And then they were asked more specifically that “how much those practices were tolerable for them”. Some other questions such as: ‘‘Can you explain it more?’’ or ‘‘is there any more to explain?’’ were proposed to the participants to make the interviews more concentrate and to focus exact on the subject.

When the participants described their experiences and no other explanations were necessary or they were repeated and superfixed for the researchers and no other new experiences were expressed, it was defined and considered as the end of the interview. The interviews lasted 45-60 minutes, audio-taped, and were transcribed verbatim. The 2^nd^ phase of the interviews was conducted 2 to 3 weeks after the first interviews had been performed.

### 2.4 Data Analysis

In data analysis for phenomenological enquiry, the researchers aimed to uncover and produce an accurate description of the phenomena being studied as experienced by the individuals.

The data analysis followed the descriptions based on Colaizzi procedural steps (1978). Defaults or pre-assumptions of the researchers resulted from their experiences, literature and previous researches were identified and excluded and literature review was postponed until after the completion of data analysis. All the interviews were carefully listened and read several times in order to extract the meanings of the participants’ descriptions and explanations. The written interviews with the women were re-matched exactly with the recorded data on tapes. All transcripts were checked against tapes for its accuracy. The credibility, conformability, auditability, dependability and transferability of the study were conducted based on Guba and Lincoln method and procedure (Alavi & Abedi, 2006). It was also achieved through confirming of the statements by the participants. The participants’ similar answer to the same question in different format or structure was the same.

Words and phrases were highlighted in the text. Concepts in the highlighted data were coded. These codes were compared to other codes within the same interview and with other interviews with different participants. Any similarities and differences were noted. Codes were grouped and categorized according to themes and then compared with concepts emerged from the research literature. On the other hand the concept(s) of any statement was explained and each concept was given a code, the codes were classified in categories and the same codes were put in the related categories and then the categories were compared with the original descriptions of the participants during the in-depth interviews for accreditation and conformity (Sanee & Nikbakht, 2004). Then the given codes and themes were checked by two psychologists. At the next stage, the results were combined as a full description from women’s experiences of giving birth and were reviewed for achieving clear and unequivocal concepts. Finally the obtained and the last outcomes were returned back to the participants for recalling and reflection to determine the accreditation and conformity of them.

## 3. Results

Data analysis developed concepts and categories. Three themes or three main concepts, some constituents and sub-themes emerged from data analysis of the women’s experiences in normal giving birth. These concepts and constituents emerged from their experiences of giving birth were conceptualized. What was found and understood as a need and seemed necessary from the concepts and the participants’ statements were as the followings:

### 3.1 Feeling Relaxation in Giving Birth Environment

This concept is broad in scale and covers a wide range of related constituents such as noise in the giving birth environment, providing privacy of women and dominant color of the environment that are components of the concept in the extracted codes. For example, one of the women told us that “Giving birth environment was very important and in an environment that noise is high and laborer hears permanent shouts of other women giving birth, her anxiety is increased”. A quiet and safe environment was the most urgent need of the most women for their relaxation, the other one mentioned. “Covering paravan and providing a private environment or private bed was necessary for the laborer, because when the environment is not quiet and people have no sense of security in their privacy, they feel embarrassed and this situation makes tolerance condition more difficult while the laborer is experiencing severe pain”, the other participant expressed. “I had the least cooperation with midwife when my environment was crowded and I lost my tolerance ability” and also unfamiliar environment with its tools was found as a factor or caused to increase the anxiety of women”, one of the other women also added: “Everything was frightening, Paints of the walls, curtains and equipments remembered us the operating room and I did not feel relax even when I had no pain”.

### 3.2 Giving Confidence to Laborer

This main concept includes two components such as support of laborer and midwife skills. The participants claimed that midwives were continually present on maternity ward and spoke in laborer language and were explaining techniques and progress of giving birth to women and this resulted in women’s peace and relaxation because of their presence in the maternity ward and they felt they were in good hands.

For example one of the participants said: “In the morning shift that I was admitted, one of the midwives performed all my needs in good morality; she even explained me how to breathe when I had pain and this caused me to tolerate pain more easily”. One of the other participants said: “Some midwives were very good and skilled at their job such as: sorting our embryo heart properly, and regulating serum drops quickly or to inform doctor if a problem came, they did painless examinations very comfortably and if I asked them any questions, they answered me willingly and well”.

### 3.3 Reduction of Stress from Unnecessary Practices (Routines)

This main concept is composed of several components such as: serum therapy, position status, pelvic examination, amniotomy and fasting. The majority of the participants discussed measures that increased their discomfort and anxiety. For example, asking one of the participants, she said: “Being in pain I liked to walk, but since I had a serum connected, I was forced to stay in bed and even I had problem for going to bathroom”. The other participant said: “As I was admitted they tore my amnion bag in an internal examination and I felt hot and fever and never felt well at all and all my clothes were wet. When I was moving, I got wet more and this caused me to stay in bed motionless, she added. “This motionlessness status was more difficult and suffering when I was in pain”. One of them claimed: “Very thirsty and hungry I was”. The other participant said:“I had no food from yesterday, I liked a little water or fruit juice to drink, but only my lips were wet with a wet gas, they were cracked”. Almost all the participants reported: Fear of pelvic examinations and discomfort resulted from it as one of the most bitter and hard experiences of themselves in giving birth”.

One of the women said: “I requested C-section (Caesarean) to escape from repeated internal examinations regularly conducted by gynecologist and midwives”. One said: “If the internal examination was not performed, giving birth was easier to tolerate”. These statements contained related codes and concepts which form infrastructures of the main concept of usual care measures after data analysis. In general women’s claims and complaints showed and appeared some new points to return us back or gave us a feedback to find new ways or solving approach to overcome challenges ahead. The findings or derived results are the map of the way for all midwives or caregivers or as a mirror that shows and reflects what and where we are and help and guide us to find and reach the goal and how we should be in the future.

## 4. Discussion

There is limited research in this area which explores and identifies women’s experiences of giving birth. However, the pain of giving birth is recognized as a large unknown process that provokes anxiety and fear. The main theoretical idea that was obtained and emerged from this study and derived directly from the data is that: If women are able to successfully adjust to their new and often unexpected reality during giving birth, and begin to reclaim their needs and their world, then they will experience a return to their normality.

However available literature related to women’s experiences of giving birth is less and often limited by its methodology, but the present study findings revealed that what experiences women obtained of giving birth phenomenon. The women, who had a giving birth, believed that providing an appropriate environment was one of the most important factors influencing the progress of giving birth. A quiet and silent environment with soft colors and preparing special environment for each woman could reduce their anxiety. In a study in accordance with the present study it was claimed that anxiety could increase secretion of catecholamine and this in turn declined oxytocin of the blood that made difficulty for the progress of giving birth (Javadnoori, & Afshari, 2008). Thus, providing a suitable environment can reduce stress and the secretion of Adrenaline, but it increases secretion of pituitary oxytocin and at the end reduces the required usage of exogenous oxytocin to the least and accelerates the progress of giving birth in this way. In Dahlberg qualitative study entitled: “women’s experience of childbirth” it was stated that most of the women wished a fast giving birth without pain. To make pain period of giving birth more tolerable and shorter can be achieved by a favorable environment (Dahlberg, Berg, & Lundgren, 1993).

Also, an article entitled “The importance of privacy in labor” performed by Judith in New Jersey of America noted that in human or animals that suffered from fear, intimidation during childbirth, catecholamine’s hormone was released that could make trouble for laborer. When mental health status of women is changed, the level of blood catecholamine is increased and this slows or stops giving birth process. This research paper offers the importance of a safe and quiet environment and warns people from unnecessary interventions and demands giving confidence, internal and inherent abilities for normal giving birth. The research also has expressed that physical and mental support must be continued, and then these findings are in accordance with our study findings and approve the results. Even research on mice has shown that when they had not any safety and privacy, their giving birth was difficult. Women will be anxious in the hospitals environments whenever they are faced with the equipments, devices and unfamiliar people due to their privacy disruption and this can lead to the release of stress hormones and make some problems for giving birth process (Judith & Lothian, 2004). Another main concept of the findings revealed from the study, was giving assurance to laborer; giving support on giving birth time was also an important and positive key factor of giving birth experience. The laborers needed support. This support can be provided by families or close relatives of those who are present in giving birth time and or by the mothers’ caregivers that are midwives, doctors and or nurses.

Waldenstrom et al study “birth experience” showed that staffs, support had a positive influence on giving birth experience. Supporting during giving birth increases the success of breast-feeding and creates strong and positive relationship between mother and child and decreases the need for intervention. The study also supports continuous presence of midwives and encouraging of laborer (Waldenstrom et al., 2007). These findings are also in accordance with our findings and emphasize midwife presence.

A qualitative study by Berg and colleagues entitled “a phenomenological study of women’s experience of giving birth with complications” showed that women’s true nature in encountering midwife and her support during giving birth is the presence of midwife, this means that cares should be performed by a midwife and the laborer status should be explained to her in her own language (Berg & Dahlberg, 1998).

Lundgren et al. in a study showed that the presence of midwife and her contact with laborer could help mothers to use their power and ability to deal with the symptoms resulted from their body (pain) during giving birth. Reduction of pain by drugs can never be a replace of communications, attentions and personnel support for the laborer (Lundgren, 1998).

Another study by Dahlberg and Berg showed that women in giving birth which are faced with midwifery complications do not state or complain with length of giving birth and medical interventions, but what is a principle and important for the laborers is midwife skills and telling them the truth ((Berg & Dahlberg, 1998). The third concept obtained from in-depth interviews with the participants was routine care measures. Women’s experiences of giving birth suggested that some common measures, including early amniotomy, starvation during giving birth, sleeping on bed and repeated pelvic examinations in giving birth were painful for them and made pain tolerance more difficult for them.

Brisson-Carroll, et al study showed that 31/2% multiparae were against amniotomy. Routine amniotomy has advantages and disadvantages that all these aspects must be considered to perform it. Amniotomy profits include reduction of giving birth length and risk of low Apgar from the fifth minute after giving birth. But there is a correlation between early amniotomy and increased rate of C-section (cesarean) because of fetal distress. So, it has been suggested that amniotomy should be done in the cases in which giving birth progress is encountered with difficulties (Brisson-Carroll et al., 1996).

Based on the study of Lundgren and Dahlberg, giving birth intervention should be used only on the base of its necessity (Lundgren, 1998). Some studies have shown that if no complications threat the mother and fetus during giving birth, mothers should eat and drink enough fluids to prevent dehydration and Ketosis (Gyte & Richens, 2006). Although vaginal examinations are a vital tool for midwifery diagnosis during giving birth, but if it is repeatedly performed, it can be uncomfortable and painful for the mothers and will increase the risk of infection (World Health Organization, 1996).

Sleeping in bed will cause pressure on the inferior vena cava and flow hypotension of uterine blood, then contraction is ineffective and giving birth will be prolonged. Ridley in his study stated that women should be encouraged during giving birth to have every position they would like to have and not to stay in bed, midwives and gynecologists should learn to do their cares and routine observations for women out of bed (Ridly, 2007).

Kordi and his colleagues in their study entitled: “The effect of maternal exposure conditions on the length of active time of first stage of labor” expressed that the length of active time of the first stage of giving birth was significantly shorter in selective situations in compare with the usual group, thus we can use different positions other than usual ones to decrease the length of staying time of laborer in hospital (Kordi, Nazari, Mansouri, & Esmaeili, 2006).

Douglas and colleagues conducted a study and found that women’s expectations could be defined as 4 main categories such as informational support, emotional support, advocacy, and competent professional care and the expectations were derived from the experiences of women who gave birth in most of researches that had been performed (Douglas, Cervin, & Bower, 2007).

Gibbins and Thomson in a study claimed that woman’s need for control of herself and circumstances is widely acknowledged and supported in the literature. A sense of control has also been found to be an important determinant of women’s satisfaction with the childbirth experience from other studies. The women in this study felt fully informed of events during labor even if they could not control them. They all felt involved in decision making and felt in control of the situation under the circumstances. All the women in this study expressed positive feelings about their labors, even though they felt that their experiences of labor were different to their expectations (Gibbins & Thomson, 2001). These findings are also in accordance with our study findings.

Mohammad Ali Beigi and her colleagues described that medical system; family participations and religious thoughts and some of the personal characteristics could decrease the pain and make it tolerable. At the same time some measures also could be a problem in labor pain and even make it worse and these factors were effective in making delivery a bad memory. These results are in accordance with the present study (Mohammad Ali Beigi, Broumandfar, Bahadoran, & Ali Abedi, 2010).

Women need to be helped and supported appropriately, especially when the reality of what they actually experience becomes clear. Then midwives need to find a proper solution or explore how care should be provided for a mother in giving birth period.

Most concepts derived from other studies showed and approved our findings on women’s experiences in the present study.

## 5. Limitations

The limitation of the study was that we selected primiparae women with normal giving birth without any giving birth-related complications who referred to state and urban health care centers. We performed a qualitative study intended to obtain the experiences of laborers. It is believed that further studies even in rural areas are needed to show and define women’s experiences from giving birth with different social and cultural backgrounds. It should even be said that more and wider studies would generate additional information about midwives roles extracted from women’s experiences; however saturation occurred in our sample.

## 6. Conclusion

Labor is considered to be one of the most painful experiences in life. The intensity of the pain experienced during labor affects maternal psychology, labor progress and fetal well-being (Shrestha, Pradhan, & Sharma, 2013).

It is important to explore women’s experiences of giving birth in order to know how they can be helped in giving birth and fulfilling it. Based on the study aim, we should mostly focus on interests, sufferings, problems and health of laborers instead of emphasis on new tools, techniques and issues such as economy, efficiency and lower cost for health care’s performance and we should consider real experiences of women giving birth to invent new methods of health cares for all the mothers. Preparation of suitable locations and facilities and getting use of labored women’s experiences can provide a situation that giving birth or this physiologic process be fulfilled with minimum intervention and in the best possible status and with enough relaxation. We should give the women giving birth the right of choice and decision-making and offer them midwifery cares at the same time and also avoid unnecessary interventions and practices to change giving birth experience to a pleasant and enjoyable experience for them and through this way reduce cesarean (C-section) rate and potential risks involved that in turn it will result in good outcomes such as breast-feeding success. Feeling a sense of control over giving birth process will help women to feel positive about their experiences, even though it may differ from their expectations. The only thing that we should do is production of knowledge on health care that can be achieved through women’s experiences in a scientific method. Then, mothers should be asked how and what giving birth is and what does it mean to them? This information can help to define the roles of gynecologists and midwives as maternity care providers.

If women are to be empowered by making choices for giving birth, then, it is therefore important for midwives to explore and discover the expectations and feelings of women in their care, and then to respect, support and fulfill them during giving birth. Then, these experiences may help the midwives to do their best for the mothers and babies since some birth places or settings are more appropriate and mother-friendly than others. These also are the best ways for birth assisters to be more mother-friendly in their dramatic task or job and the experiences can be useful in giving better care for the women. Positive and negative aspects of women giving birth should be considered to make suitable decision for the future.

At the end, we conclude that health of mothers is health of babies and they are those who will manage our future world, and then mothers should be considered any way. Educational programs and birth preparation classes for mothers would help to reduce women’s emotional discomfort and alleviate their concern for their supporters. All mothers should have the opportunity to give birth based on their own terms in a quiet, supportive and calm environment.
